# Projected costs of informal care for older people in England

**DOI:** 10.1007/s10198-023-01643-1

**Published:** 2023-12-12

**Authors:** Bo Hu, Javiera Cartagena-Farias, Nicola Brimblecombe, Shari Jadoolal, Raphael Wittenberg

**Affiliations:** Care Policy and Evaluation Centre, London School of Economics and Political Science, Clement’s Inn, London, WC2A 2AE UK

**Keywords:** Informal care costs, Economic valuation, Functional disabilities, Long-term care projections, England, I11, J11, E26, E27

## Abstract

**Background:**

Health economics research and economic evaluation have increasingly taken a societal perspective, accounting for the economic impacts of informal care. Projected economic costs of informal care help researchers and policymakers understand better the long-term consequences of policy reforms and health interventions. This study makes projections of the economic costs of informal care for older people in England.

**Methods:**

Data come from two national surveys: the English Longitudinal Study of Ageing (ELSA, *N* = 35,425) and the Health Survey for England (*N* = 17,292). We combine a Markov model with a macrosimulation model to make the projections. We explore a range of assumptions about future demographic and epidemiological trends to capture model uncertainty and take a Bayesian approach to capture parameter uncertainty.

**Results:**

We estimate that the economic costs of informal care were £54.2 billion in 2019, three times larger than the expenditure on formal long-term care. Those costs are projected to rise by 87% by 2039, faster than public expenditure but slower than private expenditure on formal long-term care. These results are sensitive to assumptions about future life expectancy, fertility rates, and progression of disabilities in the population.

**Conclusions:**

Prevention schemes aiming to promote healthy aging and independence will be important to alleviate the costs of informal care. The government should strengthen support for informal caregivers and care recipients to ensure the adequacy of care, protect the well-being of caregivers, and prevent the costs of informal care from spilling over to other sectors of the economy.

**Supplementary Information:**

The online version contains supplementary material available at 10.1007/s10198-023-01643-1.

## Introduction

Long-care care is essential to people who experience a decline in functional capability and need help with daily tasks, such as dressing, bathing, and cooking. Functional disabilities may result from the onset or progression of long-term illnesses in later life, such as dementia, stroke, cancer, multiple sclerosis, or poor mental health. In other cases, they are associated with aging and frailty. Like in many other high-income countries, people with functional disabilities in England may receive formal or informal long-term care to meet their care needs. Formal care refers to care services undertaken by paid professional staff, while the latter refers to unpaid care provided by family members, friends or neighbors.

There is mounting evidence about the projected expenditure of formal long-term care. It has been reported that the total expenditure on formal community-based care and residential care for older people in England is projected to rise by 94%, from £18.3 billion in 2018 to £35.5 billion in 2038 [1]. Meanwhile, public and private expenditure on formal long-term care in England will rise by 95% and 120%, respectively, between 2018 and 2038 [2]. However, little is known about the projected costs of informal care.

Understanding the future costs of informal care is important for three reasons. First, projected costs of informal care indicate the level of long-term care resources needed to meet people’s care needs and thus can be of great value to care planning at the national and local levels. Careful planning of long-term care resources is required to fulfill the key policy goals set out by the government of England, such as promoting personalized care, ensuring equal access to care, and reducing unmet care needs [3]. Second, a majority of long-term care tasks are undertaken by informal caregivers. The economic value of informal care is not included in GDP, but its contribution to a country’s economy should be duly recognized. The cost of informal care provides a tangible measure of caregiving activities and clarifies the important role of informal care in the long-term care system and the wider economy [4–6]. Finally, economic evaluations and cost-effective analyses are increasingly taking a societal view where the analytical lens is expanded to the costs of informal care [7–10]. Accounting for informal care costs in the projections helps researchers and policymakers obtain a deeper and more comprehensive understanding of the economic consequences of policy reforms and health interventions.

Drawing on data from two national surveys, this paper makes projections of the demand for informal long-term care for older people in England and the national costs of informal care associated with the demand. We focus on people aged 65 years and over because the future increase in demand for informal care will be mainly driven by this segment of the population. The research findings aim to contribute to two strands of academic debates in the literature: the economic valuation of informal care and the economic impacts of healthy aging.

## Costing informal care: current methodologies

Estimating the total costs of informal care involves multiplying care time by the unit costs of care. Both issues have been investigated extensively in the literature. There are different ways to measure care time. One approach is through time diaries where caregivers report the time spent on care activities as the day progresses over a certain period of time. This approach ensures the richness and the accuracy of the collected information but is time-consuming for researchers and a strain for respondents [8, 11]. Another approach is the recall method where respondents are asked to report the number of care hours in the past or a typical week. This approach is less costly than the time diary approach and thus can be more easily applied in large-scale surveys, but it is vulnerable to recall bias [12]. Furthermore, the information collected via the recall method can be sensitive to the design of the questionnaire. A survey may ask respondents to recall the total hours of informal care in one question. Alternatively, researchers can ask respondents to report care hours for individual care activities in several questions and then calculate an aggregated number. Previous research shows that the hours of care reported in the multiple-question design are higher than those based on the one-question approach [13].

Since informal care has no market price, there are ongoing debates about the best way to value its unit cost. Four commonly used approaches in the health economics literature are: the opportunity cost approach, the replacement cost approach, contingent valuation, and conjoint analysis [7]. The first two are also known as the revealed preference approach, whereas the last two are known as the stated preference approach. In the opportunity cost approach, the costs associated with giving up employment (e.g., wages) and leisure time are used to value the hourly cost of unpaid care [9, 14]. The replacement cost approach, also known as the proxy good approach, assumes that the monetary value of unpaid care is equivalent to the cost of its closest substitute such as formal home care [15]. In the contingent valuation, the unit costs are derived by assessing the amount of money respondents are willing to pay (WTP) or willing to accept (WTA) regarding one hour of unpaid care [16]. The conjoint analysis involves designing two or more hypothetical informal care situations with different attributes. The value of informal care can be derived by including money as one of the attributes and observing how respondents make choices between different situations [17].

The perspectives of measurement and valuation also matter. A study may collect information on care time from caregivers, recipients, or both. Similarly, the WTP and the WTA questions can be answered by either group of respondents [12]. It is caregivers who spend time and effort providing help, so it has been argued that studies should be conducted from their perspective. However, it is in practice challenging to separate caregiving activities from domestic activities, such as doing housework and keeping people company. In theory, only those activities that aim to meet people’s care needs count toward informal care, but neither researchers nor caregivers can ascertain them with ease. This is especially the case when caregivers and care recipients live in the same household because carrying out household tasks may be routinized within the household and their boundaries with caregiving may be blurred. Costing informal care can be further complicated by the multi-tasking of caregivers [18]. Rutherford and Bu showed that among people who reported receiving spousal care, 52.6% of them had a spouse to confirm the caregiving [19]. Urwin and colleagues investigated 1384 dyads of caregivers and care recipients who were living together [20]. They found that only 34% (*n* = 371) were mutually confirmed dyads. Among those who confirmed each other, caregivers reported providing an average of 38 h of care per week, whereas care recipients reported receiving an average of 27 h of care per week.

## Costing informal care: existing evidence

An increasing number of international studies have reported the average annual costs of informal care. A systematic review shows that the average costs varied greatly according to geographical locations and the type of illnesses under investigation [21]. Fewer studies have presented evidence on the national costs of informal care. A recent study estimated that the total annual costs of informal care among the 27 countries in the European Union ranged from €119 billion in France to €0.3 billion in Estonia [22].

Based on a literature review, we identified seven UK-wide studies estimating the national costs of informal care (Table 1). Four studies [5, 23–25] provided estimates for the general population. Three others focused on particular health conditions including dementia [14], stroke [26], and poor mental health [27]. A majority of them adopted the replacement cost approach in valuation. However, the replacement costs vary from one study to another, which led to different estimates of national costs [5, 25]. In addition, five studies took the caregivers’ perspective and two took the care recipients’ perspective.

**Table 1:** Summary of studies reporting total annual costs of informal care in the UK

Authors of study	Study perspective	Care recipient groups	Methods of valuation	Unit costs	Total annualized costs of informal care
Karlsson et al. (2006) [23]	Care recipients	Older people	Opportunity costs	£9.05 per hour	£32 billion (2000 prices)
Buckner and Yeandle (2015) [5]	Caregivers	Older people and younger adults	Replacement costs	£17.20 per hour	£132 billion (2015 prices)
Office for National Statistics (2018) [25]	Care recipients	Older people and younger adults	Replacement costs	Vary by care tasks and frequency	£68.7 billion (2016 prices)
Carers UK (2020) [24]	Caregivers	Older people and younger adults	Replacement costs	£23 per hour	£193 billion (2019 prices)
King et al. (2020) [26]	Caregivers	Older people with stroke	Replacement costs	N/A	£15.6 billion (2015 prices)
Wittenberg et al. (2019) [14]	Caregivers	People living with dementia	Replacement & opportunity costs	Vary by care tasks and employment status	£10.1 billion (2015 prices)
McDaid et al. (2022) [27]	Caregivers	People with poor mental health	Contingent valuation	£22.4 per hour	£36.4 billion (2019 prices)

While there is little UK-based evidence on the projected informal care costs for the general population, disease-specific studies show that the informal care costs for people with dementia and stroke are projected to rise by 217% [28] and 170% [26], respectively, in the next two decades. Outside the UK context, the costs of informal care for the US population with cardiovascular disease are projected to rise by 95% between 2015 and 2035 [29].

## Research methods

### Data

This study drew on data from two nationally representative surveys: The English Longitudinal Study of Ageing (ELSA) and the Health Survey for England (HSE). The ELSA follows a sample of people aged 50 and over in multiple waves and collects aging and health-related information via an interview and self-response questionnaires [30]. We used the data collected between 2012 and 2018 (waves 6 to 9). The sample size was 35,425. The HSE is a repeated cross-sectional survey [31]. A different sample is surveyed each year. A social care module for people aged 65 and over has been added to the HSE since 2011. We used data collected between 2011 and 2018. The pooled sample size was 17,292. We used the two surveys to combine their respective strengths. The longitudinal design of the ELSA enabled us to investigate the transition of care needs over time. This is essential to projections of long-term care needs. The HSE contains more detailed information than the ELSA about care needs, which improves the prediction accuracy of informal care demand and costs. In addition, we also used population, marital status, living arrangements, and mortality projections published by the Office for National Statistics (ONS) [32–34].

### Macrosimulation model

**Fig. 1:**
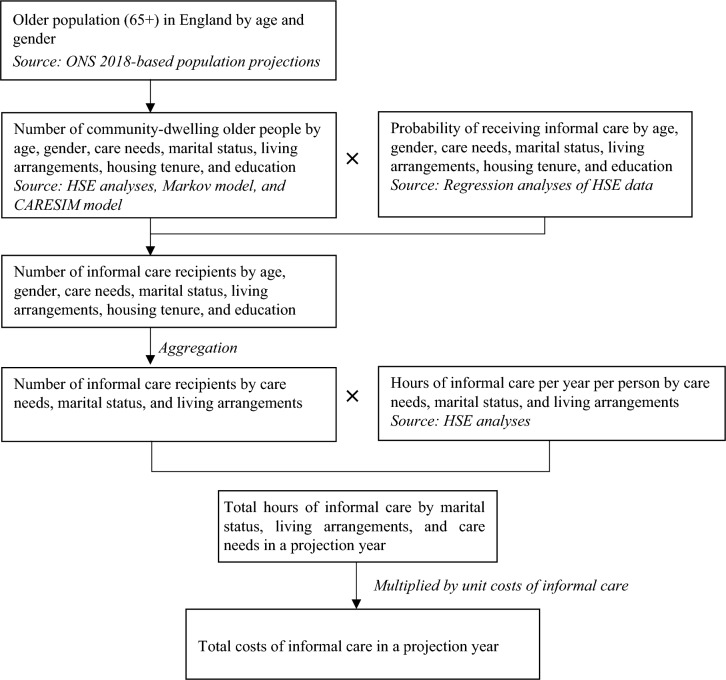
Structure of the macrosimulation model of informal care for older people

We linked a macrosimulation model with a Markov model to make projections of informal care costs. The base year of the projection model is 2019. The macrosimulation model of informal care follows the methodology developed by the Care and Policy and Evaluation Centre (CPEC, formerly known as PSSRU) [1, 35]. The model consists of three parts (Fig. 1). Drawing on the ONS population projection data and the HSE data (2011–2018), the first part divided the older population in England into 1200 subgroups according to age, gender, long-term care needs, marital status, living arrangements, housing tenure, and the number of years of full-time education. These are the factors that are strongly associated with informal care utilization in our regression analyses (Table 3). The proportions of missing values in these variables were negligible (< 0.1%). The analyses were conducted based on completed cases. We used unweighted estimates. The HSE asked respondents to report their ability to perform activities of daily living (ADLs) and instrumental activities of daily living (IADLs) on a four-point scale: ‘I can do this without help’, ‘I have difficulty doing it but can manage on my own’, ‘I can only do this with help’, and ‘I cannot do this’. People who could not perform a task without help (i.e., the latter two options) were treated as having an ADL or IADL limitation regarding this task. We created a functional disability variable with four categories to measure long-term care needs: no care needs, IADL limitations only or difficulties with ADLs (low care needs), one or two ADL limitations (medium care needs), and three or more ADL limitations (high care needs).

In the second part, we conducted regression analyses to estimate the probability of receiving informal care for people in each of the 1200 small groups. The bivariate probit regression model was used to address the issue of endogeneity attributable to the joint determination of informal care and formal home care [36]. We multiplied the number of people by the probability of informal care receipt, which gave us the number of informal care recipients in each group. Aggregating the informal care recipients led to a national estimate of informal care recipients.

The third part estimated the total hours of informal care at the national level. Since our projections focused on costs associated with care demand, we took the care recipients’ perspective. The HSE asked care recipients to report the hours of care they had received from spouses, children, relatives, friends, and neighbors in the previous week, respectively. The responses were recorded in bands (0–4 h, 5–9 h…100 + hours). Following prior research [37], we assigned the mid-values of the bands to the responses. We then added up the hours of care received from all caregivers to calculate the total hours of care for each recipient. Our regression analyses showed that, conditional upon the receipt of informal care, people with a higher level of care needs, married people, and people living with others received more hours of care. Therefore, we divided the care recipients into subgroups according to these characteristics and estimated the average hours of care for people in each subgroup. Multiplying the number of informal care recipients by the average hours of care gave us the total hours of informal care for each subgroup of the population. Aggregating the informal care hours led to a national estimate of hours of informal care for the older population.

To make sure that our projected costs of informal long-term care were directly comparable to those of formal care, we adopted the replacement cost approach to value informal care. It is estimated that the unit cost of formal home care in England was £24 per hour in 2019 prices [38], which we took as the monetary value of informal care. We multiplied the hours by the unit costs of unpaid care to estimate the national costs of unpaid care in the base year of 2019. We applied demographic, epidemiological, and socio-economic trends between 2019 and 2039 to estimate the number of informal care recipients, the total hours of informal care, and the national costs of informal care in the projection years by 2039. Projected long-term care needs were estimated by a Markov model, as described below. Our base case projections were based on a set of assumptions:

- The number of people by age and gender changes in line with the ONS 2018-based principal population projections [33].
- Marital status and living arrangements change in line with the ONS 2011-based projections [32].
- There is a constant ratio of single people living alone to single people living with their children or with others and of married people living with their partner only to married people living with their partner and others.
- Home-ownership rates for older people, as reported in the 2010/11 Family Resources Survey (FRS), change in line with projections produced by the CARESIM microsimulation model [35].
- The proportions of people receiving informal care and average hours of care remain constant in the projection years for each subgroup according to age, disability and other needs-related characteristics.
- The unit costs of home care rise in real terms with the Office for Budget Responsibility (OBR) assumptions for future trends in productivity, with an uplift in unit costs to 2024 to account for the planned rises in the national living wage [39].

### Markov model

We built a Markov model to make projections of the future prevalence of disability. There were four steps. First, drawing on the ONS population estimates, the model stratified the older population aged 45 and over by single year of age and gender in 2019. We aged the population each year. People who died exited and those who reached 65 years old entered the model. By 2039, everyone will be aged 65 years old and over. The second step involves estimating the prevalence of disability using the ELSA data (2012–2018) in 2019. The ELSA questionnaire asked respondents whether they had difficulties in performing ADLs and IADLs. We created a functional disability variable with four categories: no care needs, IADL difficulties only, one or two ADL difficulties, and three or more ADL difficulties. The unweighted estimates were used in the analyses.

**Fig. 2:**
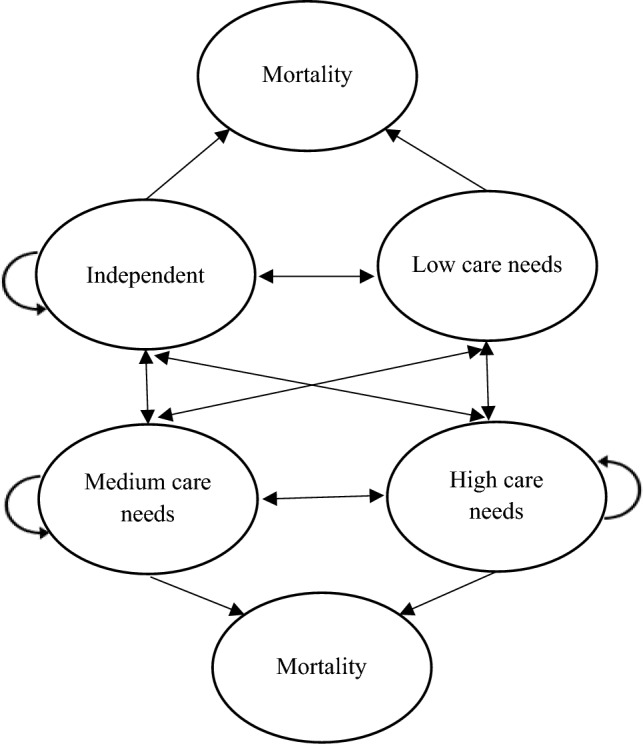
Transition of care needs in the Markov model

We took advantage of the longitudinal nature of the ELSA data to estimate the transition probabilities in the third step. A person with a certain level of disability in T may transition to any other disability status (including mortality) in T + 1 (Fig. 2). The probability of transition from T to T + 1 was allowed to vary by age, gender, and levels of disability in T. The transition probabilities were derived by fitting multinomial logistic regression models. The dependent variables were disability status in T + 1, and the independent variables were disability status, age, and gender in T. The transition probabilities were given by the predicted probabilities of the regression models. The proportions of missing values in these variables were less than 2.5%. Completed cases and unweighted estimates were used in the analyses. The mortality rates by age and gender came from the data published by the ONS [34]. The mortality rates by the levels of disability were estimated by survival analyses using data from the ELSA end-of-life survey. In the base case projections, we followed the homogeneous Markov chain assumption, which means that the transition matrices by age and gender remained constant over time.

In the final step, we aggregated the individuals to estimate the prevalence of disability by age and gender in each projection year. Our analyses showed that the prevalence of disability reported in the ELSA was largely comparable with that in the HSE. This enabled us to match the proportions of people with disability by age and gender in the Markov model with those in the macrosimulation model in the base year. We then mapped the future trends of disability projected by the Markov model onto those in the macrosimulation model and ran them through the rest of the macrosimulation model.

### Accounting for uncertainties

There are great uncertainties about informal care utilization and costs in future. Our study distinguished between model uncertainties [40] and parameter uncertainties [41]. Model uncertainties refer to those attributable to competing modeling assumptions. For example, the probability of progressing to more severe disabilities may not stay constant in the projection years but instead may slow down due to the presence of effective interventions that promote healthy aging. We investigated five alternative modeling assumptions. Incorporating the variant population projections by the ONS [42], we examined the high and low population scenarios, respectively. The high (low) population projections assume higher (lower) life expectancy, fertility rates, and migration than the ONS principal population projections. Following previous research [1], we looked at the impacts of the slow (fast) progression scenario: a reduction (increase) in the worsening of disability and an increase (reduction) in recovery from disability by 10% each year. We also investigated a scenario that assumed a constant prevalence of disability by age and gender in the projection years.

Since we were using data from random samples to make statistical inferences at the population level, the sampling distribution of the parameters had to be carefully considered. This was meant to capture parameter uncertainties. Two groups of parameters are central to our projection modeling: prevalence of disability and average hours of informal care. We adopted the Bayesian approach to derive their sampling distributions. This step was simplified by the existence of conjugate distributions [43]. Severity of disability has a generalized Bernoulli distribution, which has the Dirichlet distribution as its conjugate prior. We assumed that weekly hours of informal care would come from an exponential distribution. The conjugate prior is the Gamma distribution. The posterior distributions of disability prevalence and average hours of care are the Dirichlet distribution and the Gamma distribution, respectively, after Bayesian updating. For each run of the two linked models (i.e., Markov and macrosimulation models), we did a random draw from those sampling distributions. The two models were jointly run with 2000 repetitions, which gave us the posterior distributions of disability prevalence, the total number of care recipients, the total care hours, and the total costs in the projection years. We report the mean values and the 95% Bayesian credible intervals (CI) in this study.

## Results

Among the 17,292 older people in the HSE sample, 21% (*n* = 3539) were receiving informal care (Table 2). The 3539 informal care recipients on average received 21 h of informal care per week. Males on average received more hours of informal care than females (24 h vs. 19 h). Married people received more hours than single people. Married people included legally married couples and those who were cohabiting. People living with others received more hours than people living alone. We separately investigated the informal care recipients in the ELSA sample to externally validate these HSE results. It can be noted that the average hours of informal care for the subgroups of older people were largely consistent between the two samples (Table A1, supplementary materials).

**Table 2:** Proportion of older informal care recipients (65 +) and average number of hours of informal care per week reported in the HSE samples

	HSE 2011–2018
People receiving informal care	
No	13,753 (79.5%)
Yes	3539 (20.5%)
*N*	17,292
Average number of hours per week	
All informal care recipients	21.2
Less than 10 h	3.8
10–35 h	19.7
35–100 h	71.4
100 + h	126.2
Age	
65–74	22.5
75–84	21.0
85 +	18.9
Gender	
Male	24.0
Female	19.4
Marital status	
Single	15.2
Married	27.1
Living arrangements	
Living alone	11.5
Living with others	27.9
*N*	3539

Table 3 shows demographic, socio-economic, and health characteristics of community-dwelling older people in the HSE sample. The proportions of people with low, medium, and high care needs were 18%, 9%, and 4%, respectively (Column 3). Around one-third of older people were single and living alone, and 56% were couples living alone. In addition, 80% of the sample were owner-occupiers, and 48% had more than 16 years of full-time education. Six percent of the sample were receiving formal long-term care.

**Table 3:** Sample characteristics in HSE 2011–2018 and factors associated with informal care receipt (*N* = 17,292)

	Sample characteristics		Bivariate probit regression	
	Number	Prop. (%)	First equation	Second equation
Age				
65–69	5363	31.0		
70–74	4404	25.5	0.084 (0.048)	0.172* (0.072)
75–79	3333	19.3	0.177*** (0.049)	0.372*** (0.069)
80–84	2339	13.5	0.377*** (0.051)	0.507*** (0.07)
85 +	1853	10.7	0.566*** (0.053)	0.764*** (0.068)
Gender				
Male	7965	46.1		
Female	9327	53.9	0.253*** (0.033)	0.091* (0.043)
Level of care needs				
Independent	11,829	68.5		
Low-care needs	3142	18.2	2.293*** (0.041)	1.538*** (0.069)
Medium-care needs	1591	9.2	3.158*** (0.05)	2.083*** (0.074)
High-care needs	706	4.1	3.110*** (0.065)	2.650*** (0.083)
Marital status and living arrangements				
Single living alone	5963	34.6		
Single living with others	790	4.6	0.317*** (0.07)	–0.465*** (0.08)
Couples living alone	9605	55.7	0.06 (0.042)	–0.781*** (0.049)
Couples living with others	874	5.1	0.189* (0.081)	–0.846*** (0.124)
Housing tenure				
Owner-occupier housing	13,806	79.9		
Rented housing	3467	20.1	0.088* (0.036)	–0.018 (0.045)
Number of years of full-time education				
Less than 15 years	9004	52.2		
More than 16 years	8254	47.8	–0.112*** (0.035)	0.398*** (0.043)
Receipt of formal care				
No	16,190	93.65		
Yes	1098	6.35	–1.066*** (0.116)	
Wald *χ*^*2*^ test of ρ			*χ*^2^ (1) = 51.80***	

Outcome variable of the first equation: receipt of informal care; outcome variable of the second equation: receipt of formal care; *p < 0.05, **p < 0.01, ***p < 0.001; figures in the parentheses are standard errors.

The bivariate probit regression analyses show that people in higher age groups, females, people with a higher level of care needs, married couples, people living with other people, renters, and people who had spent less time in full-time education were more likely to receive informal care (Table 3 Column 4). The coefficients of care needs are much larger than those of other variables. People receiving formal care were less likely to receive informal care, which indicates a substitutive relationship between the two types of care.

**Fig. 3:**
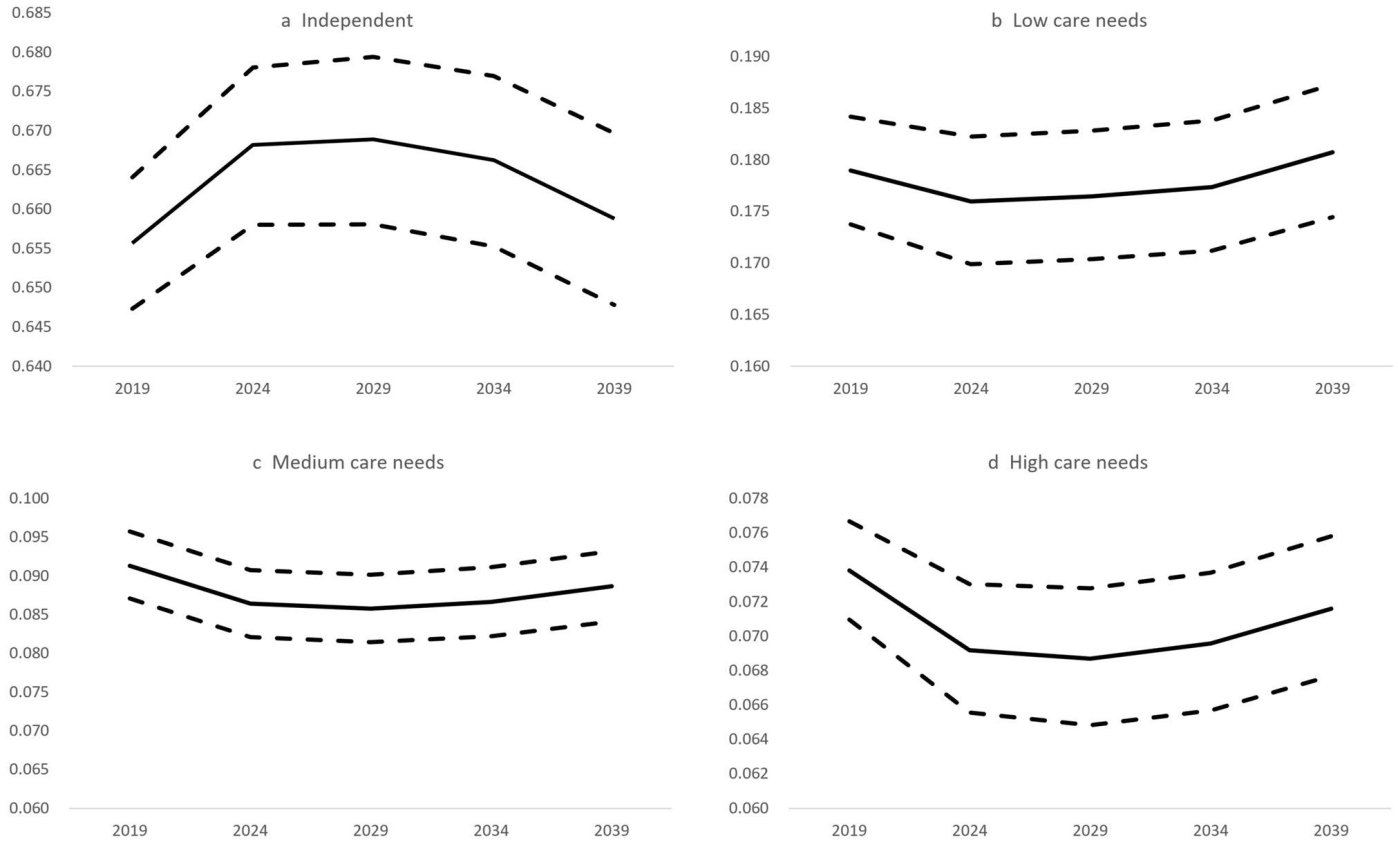
Projected prevalence of long-term care needs under the base case assumptions of the Markov model. Solid lines indicate the mean values; dashed lines indicate the 95% Bayesian credible intervals

Figure 3 reports the projected prevalence of long-term care needs deriving from the Markov model. We project that the prevalence of low care needs will decrease from 17.9% (95% CI 17.4%–18.4%) in 2019 to 17.6% in 2024 (95% CI 17.0%–18.3%) before rising to 18.1% (95% CI 17.4%–18.8%) in 2039. The prevalence of medium care needs is projected to decrease from 9.1% (95% CI 8.7%–9.6%) in 2019 to 8.9% (95% CI 8.4%–9.3%) in 2039. The prevalence of high care needs is projected to decrease from 7.4% (95% CI 7.1%–7.7%) in 2019 to 7.2% (95% CI 6.8%–7.6%) in 2039.

**Fig. 4:**
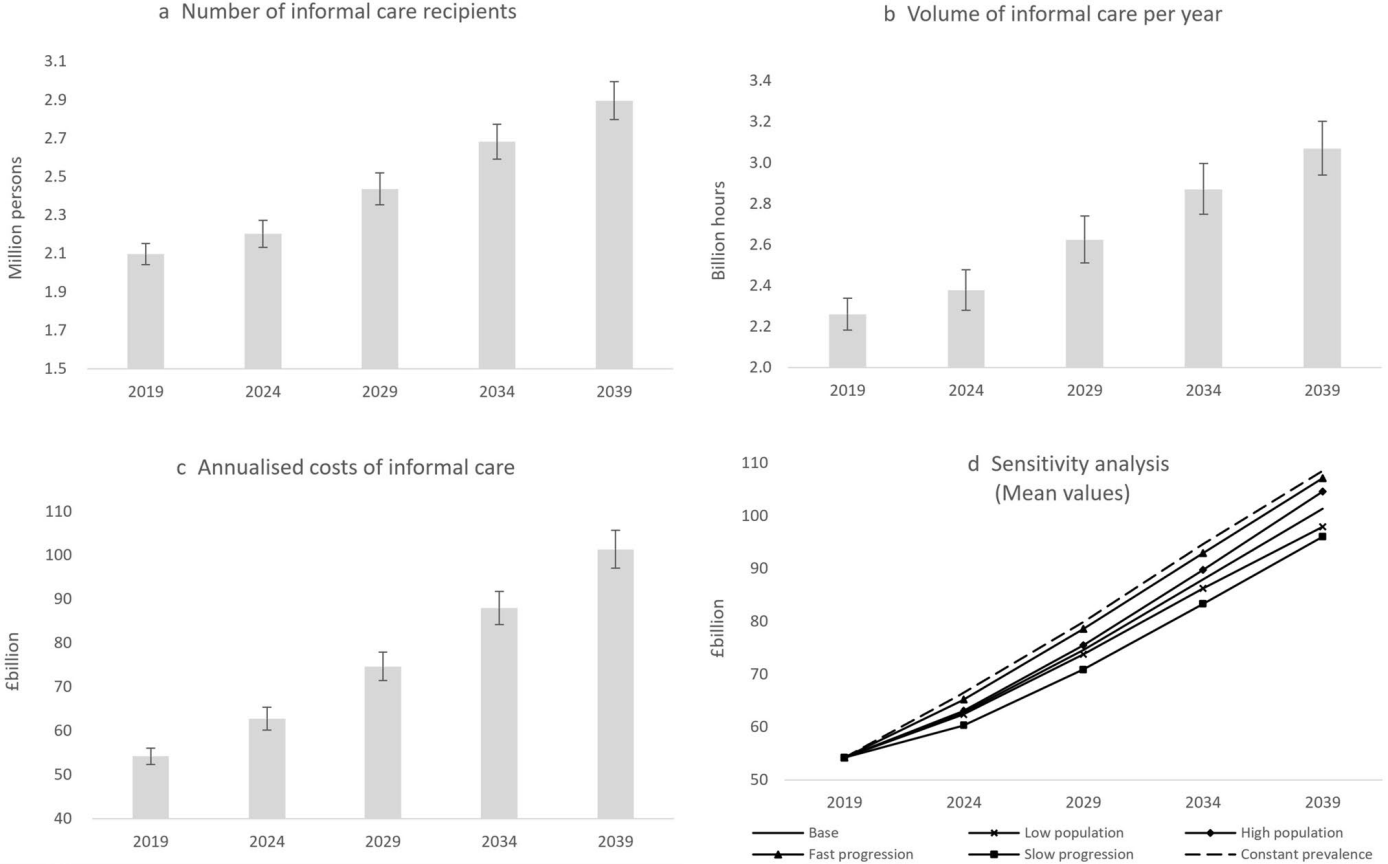
Projected number of informal care recipients and costs of informal care under the base case and alternative assumptions. 95% Bayesian credible intervals plotted on the means in panels 4a–4c

As shown in Fig. 4a, the number of older people receiving informal care was estimated to be 2.10 million (95% CI 2.04–2.15) in 2019, which is projected to rise by 37%, to 2.88 million people (95% CI 2.78–2.98) by 2039. We estimate that older people received 2.26 billion hours of informal care (95% CI 2.18–2.34) in 2019, which is projected to rise by 36%, to 3.07 billion hours (95% CI 2.94–3.20) by 2039. The total costs of informal care were estimated to be £54.2 billion (95 CI 52.6–56.5) in 2019, which are projected to rise by 87%, to £101.4 billion (95% CI 97.1–105.8) by 2039.

Table 4 breaks down the projected number of people receiving informal care and the projected costs of informal care according to marital status, living arrangements, and care needs. The number of care recipients who are single is projected to rise by more than 40% in the next two decades, faster than that of married recipients (34%). Similarly, the annualized costs of informal care are projected to rise faster among single (92–94%) than married care recipients (around 80–84%).

**Table 4:** Projected demand for and costs of informal care according to personal characteristics of informal care recipients in England

		2019			2024	2029		2034	2039	Change (%)
*Number of informal care recipients (million persons)*										
Single people										
Care from children only		0.52			0.52	0.57		0.65	0.73	41
Care from others only		0.25			0.25	0.27		0.31	0.35	42
Care from children and others		0.15			0.15	0.17		0.19	0.21	41
Married people										
Spouse care only		0.82			0.88	0.97		1.05	1.10	34
Non-spouse care only		0.14			0.15	0.17		0.18	0.19	34
Spouse care and non-spouse care		0.23			0.25	0.27		0.29	0.31	34
Care needs										
Low-care needs		1.04			1.10	1.23		1.35	1.46	40
Medium-care needs		0.76			0.77	0.85		0.94	1.02	34
High-care needs		0.30			0.31	0.34		0.38	0.40	34
*Annualized costs of informal care (£billion)*										
Marital status and living arrangements										
Single alone		12.1			13.3	15.9		19.4	23.5	94
Single with others		6.0			6.7	7.9		9.6	11.6	92
Couples living alone		33.4			39.5	47.1		54.5	61.4	84
Couples with others		2.7			3.2	3.8		4.4	4.9	80
Care needs										
Low-care needs		15.9			18.8	22.5		26.5	30.6	93
Medium-care needs		19.9			22.5	26.7		31.6	36.7	84
High-care needs		18.5			21.4	25.5		29.8	34.1	85

Figure 4d shows the projected costs of informal care under alternative modeling assumptions. Under the low and high population scenarios, the costs of informal care are projected to rise to £98.0 billion and £104.6 billion, respectively, in 2039, as opposed to £101.4 billion under the base case assumptions. Under the slow and fast progression scenarios, the costs of informal care are projected to rise to £96.1 billion and £107.1 billion, respectively, in 2039. Under the assumption of the constant prevalence of care needs according to age group and gender, the prevalence of care needs in the overall population aged 65 years old and over is projected to rise from 34.5% to 36.2% (Table 5). This is notably higher than the projected prevalence in our base case. In the constant prevalence scenario, the costs of informal care are projected to rise to £108.5 billion in 2039, as opposed to £101.4 billion under the base case assumptions (Fig. 4d).

**Table 5:** Projected prevalence of long-term care needs in the older population under alternative assumptions

	2019	2024	2029	2034	2039
Base case assumptions	34.5%	32.9%	32.9%	33.1%	33.9%
Increased progression (10% per year)	34.5%	33.9%	34.2%	34.5%	35.3%
Decreased progression (10% per year)	34.5%	32.0%	31.6%	31.8%	32.5%
Constant prevalence by age and gender	34.5%	34.9%	35.2%	35.6%	36.2%

## Discussion

Drawing on data from two nationally representative surveys spanning nearly a decade, this study made projections of the demand for and costs of informal care for older people aged 65 years old and over in England. Based on a Markov model, we projected that the prevalence of low care needs in the population will decrease in the next decade before rising back to the current level by 2039. Meanwhile, we projected an overall downward trend in the prevalence of medium and high care needs in the next two decades. The trends derived from our Markov model are broadly consistent with those reported by the Population Ageing and Care microsimulation model (PACSim) (Figure A1, supplementary materials), despite the differences between the two models in terms of the methodologies and the definitions of long-term care needs [1].

Some of the existing studies have assumed that the prevalence of care needs according to age and gender will remain constant in future [2, 35]. The ONS projected that the proportion of older people in higher age groups will continue to rise. Meanwhile, the prevalence of care needs is higher among people in higher age groups. This results in an overall rising prevalence of care needs in the older population under the constant prevalence assumption. In comparison, our Markov model assumes constant probabilities in transitions of care needs according to age and gender. Such an assumption accounts for the temporal changes in a person’s care needs status and leads to more optimistic projections of the prevalence of care needs in future. Indeed, even our most pessimistic scenario (i.e., fast progression of care needs) leads to more optimistic projections than those under the constant prevalence assumption (Table 5).

We estimated that people aged 65 and over received a total of 2.26 billion hours of informal care in 2019. Using data from the Family Resources Survey (FRS), the ONS studies reported that adults in the United Kingdom received 7.9 billion hours of informal care, among which around 2.79 billion hours of care (35.3%) were received by people aged 70 and over [25]. Given that England’s population is 84% of that of the UK, this translates into approximately 2.34 billion hours of informal care for care recipients aged 70 and over in England. Our analyses of the HSE data show that, among recipients aged 65 and over, 80% of care hours were received by people aged 70 and over. Extrapolating the ONS estimates to care recipients aged 65 and over leads to 2.93 billion hours of informal care (i.e., 2.34/0.8), which is higher than our estimate of 2.26 billion hours. One possible reason for this difference is that the FRS participants who reported receiving continuous help were assumed in the ONS studies to receive 168 h of care per week. Arguably, the ONS figures represent the upper bounds of the estimated hours of informal care.

The total hours of informal care will need to increase by nearly 40% in the following two decades to keep up with the demographic and epidemiological trends in the older population. The rise in care demand will be uneven among different groups of people. In particular, it is projected to rise faster among single than married people. This is because the majority of single people aged 65 and over are widowed and the chances of widowhood rise with age. As the proportion of people in higher age groups increases, so will that of widowed people in the population. It is worth noting that widowed people do not have their spouses to provide care and rely on non-spouse care to meet their needs. Therefore, the demand for care from children and other family members is projected to rise faster than that from spouses.

Our study on informal long-term care followed the same economic assumptions as previous studies regarding projections of formal long-term care [1, 2]. This was to make sure that the contribution of informal care to the economy was not only quantifiable but also comparable to that of formal care. Based on the replacement costs approach and taking a care recipient perspective, the monetary value of informal care for older people aged 65 and over is estimated to have been £54.2 billion in 2019 (2019 prices). This is about three times larger than the total expenditure on formal residential and community care (£19.5 billion, 2019 prices) and ten times larger than the expenditure on formal community care (£5.4 billion, 2019 prices). The costs of informal care are projected to increase to £101.4 billion by 2039, a rise of 87%. Informal care costs are projected to rise faster than public expenditure (84%) but slower than private expenditure on formal care (94%) [1].

Our projected costs of informal care are sensitive to assumptions about future life expectancy, fertility rates, and progression of disabilities. A 10% decrease in the probability of progressing to more severe functional disabilities per year will result in a decrease of £5.3 billion in the costs of informal care by 2039 in comparison to the projections under the base case assumptions. A 10% increase in the transition probabilities will increase the costs of informal care by £5.8 billion by 2039. These results suggest that it is important for the government to develop effective intervention and prevention schemes to delay the onset and progression of care needs for the older population. Not only will this contribute to the well-being of older people and a healthy aging society, but it will also have important economic benefits. It will relieve caregivers from caregiving responsibilities and enable them to engage in other productive or life activities of their own choosing.

Our projections in the base case scenario assumed that a rise in the supply of informal care could keep up with demand. Such an assumption enabled us to focus on the demand side drivers in the projections and investigate the level of resources needed to fully meet the demand for care. However, given population aging and rising multi-morbidity, it is unlikely that the supply of informal care will keep up with demand. Brimblecombe and colleagues projected that the number of informal caregivers will increase by 16% in the next two decades [44]. This is much lower than our projected rise in demand for informal care in the older population (37%).

The patterns of long-care utilization will have to change markedly to adapt to the looming care gap. There will be three possible scenarios. First, the level of care provided to older people may be inadequate. Some people, especially those with mild functional limitations, may choose, or be able, to cope with their care needs on their own, while others may be left with unmet care needs. Second, caregivers may need to increase the provision of care. A caregiver may need to provide more hours of informal care or provide care to more recipients. The UK Census data show that the proportion of people in England and Wales providing 20 to 49 h of unpaid care a week increased from 1.5% to 1.9% and the proportion of people providing 50 or more hours of unpaid care increased from 2.7% to 2.8% between 2011 and 2021 [45]. Such a trend may need to continue in future. Finally, there will need to be a lowering of the threshold to access government-funded support and a more integrated care system. This is especially important when the spouse of a care recipient passes away and the care responsibility needs to be taken up by adult children. An increase in the supply of formal care means that the responsibility can be shared between adult children and professional caregivers [46]. In this scenario, the costs of formal care are projected to rise further, but the projected increase in informal care costs will be blunted. The reality will likely be a mixture of those three possible scenarios. Their relative balance will be the result of interactions and negotiations between the state, the private market, and the family within the mixed economy of care as well as within families.

The re-configuration of patterns of care will not be cost-free, however. Studies have reported that unmet needs can lead to an accelerated progression of mental health problems and functional disabilities, which in turn incurs health and long-term care costs [47, 48]. In addition, the international literature has shown that intensive caregiving can have negative consequences for caregivers’ mental health or force caregivers to leave employment, which is associated with costs for mental health care and a loss of productivity or earnings [49–52]. Those negative impacts and associated costs may have a tipping point that compromises the sustainability of informal caregiving in the long run. Moreover, major concerns are raised about the well-being of working-age caregivers providing care to older parents, as our projections suggest that they will face a faster rise in care demand than spouse caregivers and some of them also have other life responsibilities to fulfill such as providing care to young children and/or paid employment. In anticipation of increased demand, the readiness of children to provide care for their parents may change, especially in the current climate of the cost-of-living crisis and falling property values. All of these issues point to the ‘spillover effect’ of informal caregiving on other sectors of the economy. As such, the important role of the government in ensuring the adequacy of care for recipients and protecting the economic security and well-being of informal caregivers comes to the fore. To steer the mixed economy of care, the government may also want to strengthen the evidence base on assisted living devices and invest in technologies that can boost the self-caring capability of older people and the caregiving capacity of informal caregivers.

Building upon the existing literature on the economic valuation of informal care, our study is among the first to report the projected economic costs of informal care for older people. The multi-dataset multi-model approach adds considerable strength to the study. Meanwhile, the limitations of the study should be acknowledged. First, our study focused on the self-reported direct labor input of informal caregivers when costing informal care. Indirect costs associated with caregiving such as negative consequences for caregivers’ health or loss of productivity were not quantified. Second, our projections are based on a series of assumptions about future demographic, epidemiological, and socio-economic trends. Like all projection models, making these assumptions inevitably involves a degree of subjectivity. Our sensitivity analyses aimed to mitigate this limitation. Finally, our projections did not consider the impacts of major events, such as Brexit and COVID-19 pandemic. Although there are reports that the provision of formal and informal care has changed notably (e.g., a shortage of formal caregivers and rising mental distress of informal caregivers) in the wake of these events, it is unclear at the time of writing whether such a change is temporary or will persist in the long run.

## Conclusion

The costs of informal care for older people in England are projected to increase substantially by 2039. The increase in demand for informal care will be uneven among different groups of care recipients. Caregivers are likely to face mounting challenges to keep up with the rise in care demand, putting their own well-being at risk, and unmet needs may increase. Measures taken by the government to delay the onset and progression of care needs would help to offset those challenges and protect the well-being of older people. It is equally important for the government to constantly monitor and appraise how care needs are likely to change in the population, make plans early, and make sure that adequate care and high-quality support are delivered to those who need them. This will prevent the costs of informal caregiving from spilling over to other sectors of the economy.

### Supplementary Information

Below is the link to the electronic supplementary material.

Supplementary file1 (DOCX 255 KB)

## Data Availability

All of the data used in this study are publicly available. The programming code developed for data analyses and model construction will be available upon request.
