# Supplementary material for: Projected costs of informal care for older people in England
Source: Eur J Health Econ. 2023 Dec 12;25(6):1057–70. doi: 10.1007/s10198-023-01643-1 (PMC11283415; doi:10.1007/s10198-023-01643-1)
Supplement: Supplementary file 1 — Supplementary file1 (DOCX 255 KB) [file 10198_2023_1643_MOESM1_ESM.docx]

**Appendix**

Table A1 Comparing informal care recipients and care hours in the HSE and ELSA samples

|  | **HSE 2011-2018** | **ELSA 2012-2018 (waves 6-9)** |
| --- | --- | --- |
| **People receiving informal care** |  |  |
| No | 13,753 (79.5%) | 17,801 (81.5%) |
| Yes | 3,539 (20.5%) | 4,014 (18.5%) |
| **N** | 17,292 | 21,815 |
| **Average number of hours per week** |  |  |
| All informal care recipients | 21.2 | 20.0 |
| Less than 10 hours | 3.8 | 3.7 |
| 10-35 hours | 19.7 | 20.5 |
| 35-100 hours | 71.4 | 72.6 |
| 100+ hours | 126.2 | 125.5 |
| Age |  |  |
| 65-74 | 22.5 | 18.6 |
| 75-84 | 21.0 | 20.5 |
| 85+ | 18.9 | 21.2 |
| Gender |  |  |
| Male | 24.0 | 23.9 |
| Female | 19.4 | 17.8 |
| Marital status |  |  |
| Single | 15.2 | 16.2 |
| Married | 27.1 | 23.0 |
| Living arrangements |  |  |
| Living alone | 11.5 | 11.0 |
| Living with others | 27.9 | 24.0 |
| **N** | 3,539 | 4,014 |

Table A2 Comparing unweighted and weighted estimates of informal care recipients and care hours in the HSE sample (N=17,292)

|  | **Unweighted estimates** | **Weighted estimates** |
| --- | --- | --- |
| **Proportion of care recipients** |  |  |
| No | 79.5% | 79.4% |
| Yes | 20.5% | 20.6% |
| **Average number of hours per week** |  |  |
| All informal care recipients | 21.2 | 21.3 |
| Less than 10 hours | 3.8 | 3.8 |
| 10-35 hours | 19.7 | 19.7 |
| 35-100 hours | 71.4 | 71.1 |
| 100+ hours | 126.2 | 126.5 |
| Age |  |  |
| 65-74 | 22.5 | 22.9 |
| 75-84 | 21.0 | 21.3 |
| 85+ | 18.9 | 19.3 |
| Gender |  |  |
| Male | 24.0 | 24.3 |
| Female | 19.4 | 19.8 |
| Marital status |  |  |
| Single | 15.2 | 15.7 |
| Married | 27.1 | 27.4 |
| Living arrangements |  |  |
| Living alone | 11.5 | 11.6 |
| Living with others | 27.9 | 28.2 |

Figure A1 A comparison of projected prevalence of long-term care needs reported by PACSim microsimulation model and Markov model under the base case assumptions


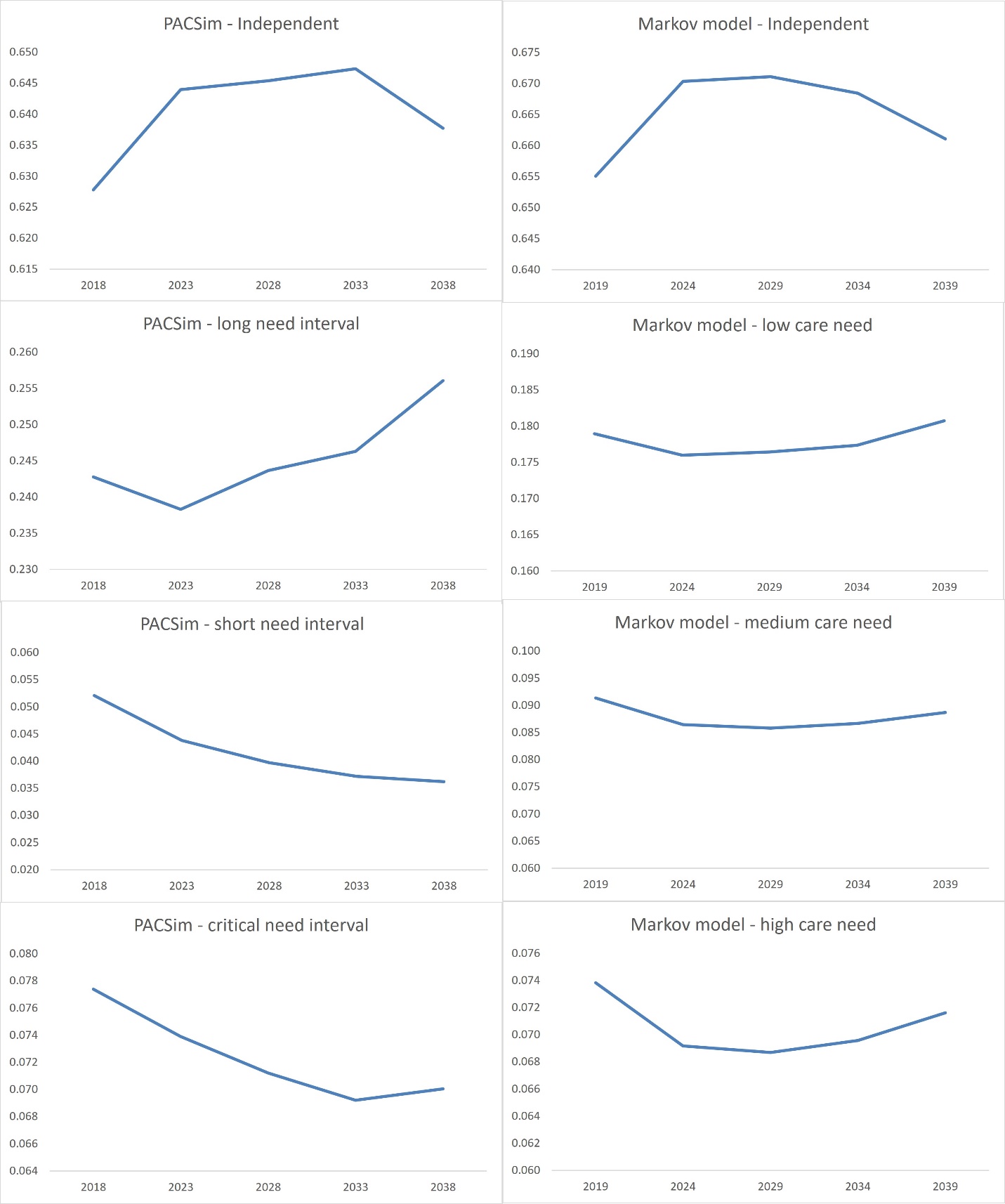


Notes: Projected prevalence of care needs in the PACSim model is calculated based on results reported in Appendix Table 1 in Kingston et al. (2022)
